# Supplementary material for: A Panax quinquefolius-Based Preparation Prevents the Impact of 5-FU on Activity/Exploration Behaviors and Not on Cognitive Functions Mitigating Gut Microbiota and Inflammation in Mice
Source: Cancers (Basel). 2022 Sep 10;14(18):4403. doi: 10.3390/cancers14184403 (PMC9496716; doi:10.3390/cancers14184403)
Supplement: Supplementary file 1 [file cancers-14-04403-s001.zip › cancers-1852687-supplementary.pdf]

cancers

MDPI

Supplementary tables

A *Panax quinquefolius*-based preparation prevents the impact of 5-FU on activity/exploration behaviors and not on cognitive functions *via* gut microbiota and inflammation in mice

Renaud Parment, Martine Dubois, Laurence Desrues, Alexandre Mutel, Kléouforo-Paul Dembélé, Nicolas Belin, Laure Tron, Moïse Coëffier, Vincent Compère, Céline Féger, Florence Joly, Pascal Hilber, David Ribet and Hélène Castel

Supplementary tables

Table S1. Primer sequences used to quantify intestinal bacteria.

Taxon	Forward	Reverse	T an [a]	Ref.
Archaea	GYGCASCAGKCGMGAAW	TTACCGCGGCKGCTG	48.5	[1]
Eubacteria	ACTCCTACGGGAGGCAGCAG	ATTACCGCGGCTGCTGG	60	[2]
Firmicutes	ATGTGGTTTAATTCGAAGCA	AGCTGACGACAACCATGCAC	51	[3]
Bacteroidetes	CATGTGGTTTAATTCGATGAT	AGCTGACGACAACCATGCAG	51	[3]
Betaproteobacteria	GGGGAATTTTGGACAATGGG	ACGCATTTCACTGCTACACG	58	[4]
Gammaproteobacteria	CMATGCCGCGTGTGTGAA	ACTCCCCAGGCGGTCDACTTA	54	[4]
Deltaproteobacteria	GGTGTAGGAGTGAARTCCGT	TACGTGTGTAGCCCTRGRC	62	[5]
Verrucomicrobia	GAATTCTCGGTGTAGCA	GGCATTGTAGTACGTGTGCA	59	[5]
Lactobacillus spp.	AGCAGTAGGGAATCTTCCA	CACCGCTACACATGGAG	58	[6]
L. reuteri	ACCGAGAACACCGCGTTATTT	ACCTAAACAATCAAAGATTGTCT	59	[6]
L. murinus/animalis	TCGAACGAAACTTCTTTATCACC	CGTTCGCCACTCAACTCTTT	60	[6]
L. johnsonii/gasseri	CACTAGACGCATGTCTAGAG	AGTCTCTCAACTCGGCTATG	60	[6]
L. acidophilus	CCTTTCTAAGGAAGCGAAGGAT	AATTCTCTTCTCGGTCGCTCTA	58	[6]

Note. [a] : Temperature of annealing

Table S2. Summary of the behavioral phenotypes of NaCl vs 5-FU mice treated with placebo, vitamin C or Qiseng®.

Spontaneous activity and exploration	Variable	Mean ± SEM	t_{DF} or Mean rank diff. (*r*)	*P* value
Open field test				
NaCl/placebo vs. 5-FU/placebo	Total distance	36.66 ± 2.369 vs. 10.79 ± 2.440	t_{60} = 5.237	******P* < 0.001**
	Total time immobile	156.8 ± 15.18 vs. 433.2 ± 32.09	*r* = -31.61	*****P* = 0.001**
	Vertical activity	59.64 ± 5.137 vs. 16.75 ± 3.985	t_{60} = 5.704	******P* < 0.001**
	Time in center	144.2 ± 16.18 vs. 59.31 ± 14.66	*r* = 23.66	****P* = 0.05**
	Distance in center	11.99 ± 1.040 vs. 3.323 ± 0.7842	*r* = 32.69	******P* < 0.001**
	Time in periphery	455.8 ± 16.18 vs. 540.7 ± 14.66	*r* = -23.62	****P* = 0.05**
	Distance in periphery	20.67 ± 1.693 vs. 7.463 ± 1.773	t_{60} = 4.444	******P* < 0.001**
NaCl/vitamin C vs. 5-FU/vitamin C	Total distance	36.60 ± 2.602 vs. 17.87 ± 4.346	t_{60} = 4.284	*****P* = 0.001**
	Total time immobile	117.8 ± 11.55 vs. 313.2 ± 55.44	*r* = -27.70	****P* = 0.01**
	Vertical activity	58.60 ± 7.305 vs. 22.36 ± 6.194	t_{60} = 4.604	******P* < 0.001**
	Time in center	141.6 ± 11.41 vs. 141.3 ± 40.38	*r* = 10.15	*P* > 0.99
	Distance in center	13.22 ± 0.8219 vs. 7.411 ± 1.811	*r* = 26.49	****P* = 0.02**
	Time in periphery	458.4 ± 11.41 vs. 458.7 ± 40.38	*r* = -10.20	*P* > 0.99
	Distance in periphery	23.38 ± 2.235 vs. 10.46 ± 2.817	t_{60} = 4.156	*****P* = 0.002**
NaCl/Qiseng® vs. 5-FUQiseng®	Total distance	28.71 ± 2.062 vs. 32.91 ± 3.411	t_{60} = 0.9801	*P* > 0.99
	Total time immobile	205.6 ± 24.22 vs. 169.4 ± 21.94	*r* = 7.517	*P* > 0.99
	Vertical activity	54.70 ± 4.863 vs. 56.08 ± 5.013	t_{60} = 0.1794	*P* > 0.99
	Time in center	169 ± 13.80 vs.	*r* = 5.433	*P* > 0.99

		150.8 ± 12.83		
	Distance in center	10.28 ± 0.6599 vs. 11.35 ± 1.196	$r = -2.067$	$P > 0.99$
	Time in periphery	431 ± 13.80 vs. 449.2 ± 12.83	$r = -5.433$	$P > 0.99$
	Distance in periphery	18.43 ± 1.557 vs. 21.57 ± 2.413	$t_{60} = 1.028$	$P > 0.99$
Actimetry				
NaCl/placebo vs. 5-FU/placebo	Activity light phase	271.9 ± 18.58 vs. 212.7 ± 16.76	$t_{62} = 2.159$	$P = 0.48$
	Activity dark phase	1062 ± 42.42 vs. 753.3 ± 38.26	$t_{62} = 5.121$	*****$P < 0.001$**
NaCl/vitamin C vs. 5-FU/vitamin C	Activity light phase	273.6 ± 24.62 vs. 297.0 ± 26.08	$t_{62} = 0.8532$	$P > 0.99$
	Activity dark phase	1038 ± 53.05 vs. 814.0 ± 41.97	$t_{62} = 3.710$	****$P = 0.004$**
NaCl/Qiseng® vs. 5-FU/Qiseng®	Activity light phase	260.3 ± 13.43 vs. 236.1 ± 12.75	$t_{62} = 0.8820$	$P > 0.99$
	Activity dark phase	920.9 ± 34.68 vs. 969.7 ± 43.40	$t_{62} = 0.8089$	$P > 0.99$
Light/dark box				
NaCl/placebo vs. 5-FU/placebo	Nb of headdip	16.09 ± 1.492 vs. 4.917 ± 0.8207	$t_{61} = 5.563$	*****$P < 0.001$**
	Light zone entries	10.55 ± 0.8567 vs. 1.000 ± 0.5365	$r = 33.74$	*****$P < 0.001$**
	Time in the light zone	69.06 ± 8.930 vs. 4.750 ± 5.866	$r = 31.78$	****$P = 0.001$**
	Vertical activity	2.727 ± 0.8538 vs. 0.1667 ± 0.1667	$r = 20.53$	$P = 0.12$
NaCl/vitamin C vs. 5-FU/vitamin C	Nb of headdip	17.09 ± 1.729 vs. 8.636 ± 1.707	$t_{61} = 4.121$	****$P = 0.002$**
	Light zone entries	10.91 ± 1.282 vs. 2.545 ± 0.8776	$r = 27.73$	***$P = 0.01$**
	Time in the light zone	73.27 ± 8.790 vs. 13.31 ± 5.866	$r = 26.73$	***$P = 0.02$**
	Vertical activity	3.909 ± 1.268 vs. 0.5455 ± 0.3123	$r = 20.14$	$P = 0.16$
NaCl/Qiseng® vs.	Nb of headdip	16.70 ± 1.739 vs. 15.58 ± 1.090	$t_{61} = 0.5120$	$P > 0.99$

5-FU/Qiseng®	Light zone entries	12.60 ± 1.384 vs. 9.000 ± 1.723	$r = 11.69$	$P > 0.99$
	Time in the light zone	109.6 ± 15.66 vs. 66.60 ± 16.20	$r = 13.84$	$P > 0.99$
	Vertical activity	8.500 ± 2.814 vs. 3.833 ± 2.014	$r = 20.10$	$P = 0.17$
**Anxiety-like behaviors**				
Elevated plus maze				
NaCl/placebo vs. 5-FU/placebo	Total distance	5.417 ± 0.5636 vs. 3.443 ± 0.4558	$t_{61} = 2.848$	$P = 0.09$
	Total time immobile	174.8 ± 10.82 vs. 215.2 ± 10.97	$t_{61} = 2.367$	$P = 0.32$
	SAP	8.818 ± 1.536 vs. 5.333 ± 1.003	$t_{61} = 2.518$	$P = 0.22$
	Head dips	12.64 ± 1.830 vs. 17.38 ± 1.248	$t_{61} = 1.770$	$P > 0.99$
	% of time in open arms	14.73 ± 2.800 vs. 10.17 ± 5.452	$r = 19.45$	$P = 0.25$
	% of distance crossed in open arms	13.55 ± 2.372 vs. 10.83 ± 3.774	$r = 9.235$	$P > 0.99$
NaCl/vitamin C vs. 5-FU/vitamin C	Total distance	4.219 ± 0.4902 vs. 3.588 ± 0.4329	$t_{61} = 0.8909$	$P > 0.99$
	Total time immobile	189.7 ± 15.19 vs. 199.7 ± 11.78	$t_{61} = 0.5691$	$P > 0.99$
	SAP	3.545 ± 0.6518 vs. 3.545 ± 0.8672	$t_{61} = 0.0$	$P > 0.99$
	Head dips	7.545 ± 1.479 vs. 5.818 ± 1.271	$t_{61} = 0.8590$	$P > 0.99$
	% of time in open arms	8.364 ± 1.795 vs. 9.091 ± 1.979	$r = -1.227$	$P > 0.99$
	% of distance crossed in open arms	10.00 ± 2.212 vs. 11.64 ± 2.387	$r = -2.818$	$P > 0.99$
NaCl/Qiseng® vs. 5-FU/Qiseng®	Total distance	5.403 ± 0.5909 vs. 4.432 ± 0.4556	$t_{61} = 1.365$	$P > 0.99$
	Total time immobile	187.2 ± 12.27 vs. 185 ± 11.93	$t_{61} = 0.1286$	$P > 0.99$
	SAP	8.3 ± 1.106 vs. 6.917 ± 0.9728	$t_{61} = 1.035$	$P > 0.99$
	Head dips	8.3 ± 1.961 vs.	$t_{61} = 0.0660$	$P > 0.99$

		8.167 ± 1.230		
	% of time in open arms	11.20 ± 3.172 vs. 21.50 ± 7.202	r = -8.550	P > 0.99
	% of distance in open arms	12.80 ± 3.140 vs. 21.92 ± 4.137	r = -13.47	P > 0.99
Anhédonia test				
NaCl/placebo vs. 5-FU/placebo	Sucrose preference (Day 3 - Week 5)	73.27 ± 3.756 vs. 64.08 ± 2.569	t_{64} = 1.995	P = 0.72
	Sucrose preference (Day 4 - Week 5)	72.18 ± 3.695 vs. 66.42 ± 3.220	t_{64} = 1.252	P > 0.99
NaCl/vitamin C vs. 5-FU/vitamin C	Sucrose preference (Day 3 - Week 5)	69.42 ± 2.327 vs. 72.92 ± 2.190	t_{64} = 0.7771	P > 0.99
	Sucrose preference (Day 4 - Week 5)	72 ± 2.579 vs. 76.33 ± 2.112	t_{64} = 0.9621	P > 0.99
NaCl/Qiseng® vs. 5-FU/Qiseng®	Sucrose preference (Day 3 - Week 5)	69.36 ± 3.374 vs. 65.75 ± 3.512	t_{64} = 0.7847	P > 0.99
	Sucrose preference (Day 4 - Week 5)	69.64 ± 3.323 vs. 65.83 ± 5.057	t_{64} = 0.8258	P > 0.99
**Depressive-like behaviors**				
Tail suspension test				
NaCl/placebo vs. 5-FU/placebo	Immobility duration	180.9 ± 7.503 vs. 192.2 ± 12.99	r = -10.45	P > 0.99
	Latency to the first immobile episode	60.57 ± 7.779 vs. 53.77 ± 6.827	t_{60} = 0.7075	P > 0.99
NaCl/vitamin C vs. 5-FU/vitamin C	Immobility duration	192.5 ± 5.810 vs. 207.7 ± 7.432	r = -8.955	P > 0.99
	Latency to the first immobile episode	66.98 ± 5.890 vs. 52.01 ± 8.619	t_{60} = 1.558	P > 0.99
NaCl/Qiseng® vs. 5-FU/Qiseng®	Immobility duration	185.5 ± 7.888 vs. 194.9 ± 12.17	r = -1.908	P > 0.99
	Latency to the first immobile episode	60.87 ± 5.960 vs. 59.35 ± 5.163	t_{60} = 0.1575	P > 0.99
Forced swim test				
NaCl/placebo vs. 5-FU/placebo	Immobility duration	245.5 ± 8.928 vs. 241.3 ± 6.072	t_{61} = 0.3252	P > 0.99
	Latency to the first immobile episode	58.95 ± 11.06 vs. 69.02 ± 8.964	r = -6.939	P > 0.99
NaCl/vitamin C vs.	Immobility duration	238.5 ± 8.312 vs. 240.6 ± 12.25	t_{61} = 0.1617	P > 0.99

5-FU/vitamin C	Latency to the first immobile episode	67.82 ± 5.330 vs. 89.05 ± 13.36	*r* = -8.955	*P* > 0.99
NaCl/Qiseng® vs. 5-FU/Qiseng®	Immobility duration	221 ± 10.75 vs. 226.8 ± 9.910	t_{61} = 0.4243	*P* > 0.99
	Latency to the first immobile episode	92.13 ± 10.44 vs. 81.91 ± 7.029	*r* = 6.575	*P* > 0.99
**Spatial learning and memory**				
Morris Water Maze				
NaCl/placebo vs. 5-FU/placebo	**Initiation – D8**			
	Escape latency	42.15 ± 3.490 vs. 46.19 ± 2.834	*r* = -11.11	*P* > 0.99
	Distance	6.196 ± 0.6418 vs. 6.863 ± 0.5011	*r* = -10.72	*P* > 0.99
	Mean speed	0.1334 ± 0.0099 vs. 0.1393 ± 0.0062	*r* = 3.625	*P* > 0.99
	**Latency (Learning)**			
	Day 9	43.31 ± 3.454 vs. 45.31 ± 2.886	t_{61} = 0.5139	*P* > 0.99
	Day 10	29.38 ± 2.854 vs. 42.29 ± 2.685	t_{61} = 3.318	***P* = 0.01**
	Day 11	23.42 ± 2.764 vs. 31.82 ± 2.811	t_{61} = 2.158	*P* = 0.47
	Day 12	10.97 ± 1.253 vs. 19.81 ± 2.404	t_{61} = 2.270	*P* = 0.35
	**Distance (Learning)**			
	Day 9	5.957 ± 0.6114 vs. 6.683 ± 0.5303	t_{61} = 1.009	*P* > 0.99
	Day 10	4.067 ± 0.4789 vs. 5.364 ± 0.4497	t_{61} = 1.802	*P* > 0.99
	Day 11	3.783 ± 0.4677 vs. 4.390 ± 0.4178	t_{61} = 0.8436	*P* > 0.99
	Day 12	1.691 ± 0.2432 vs. 2.902 ± 0.4452	t_{61} = 1.682	*P* > 0.99
	**Speed (Learning)**			
	Day 9	0.1331 ± 0.008 vs. 0.1425 ± 0.0057	t_{61} = 0.9114	*P* > 0.99
	Day 10	0.1366 ± 0.008 vs. 0.1245 ± 0.0063	t_{61} = 1.185	*P* > 0.99
	Day 11	0.1522 ± 0.0086 vs.	t_{61} = 1.445	*P* > 0.99

	0.1374 ± 0.0067		
Day 12	0.1433 ± 0.0071 vs. 0.1405 ± 0.0074	$t_{61} = 0.2805$	$P > 0.99$
**Probe test – D12**			
% of time spent in NW quadrant	38.45 ±2.042 vs. 37.75 ± 3.119	$t_{61} = 0.1188$	$P > 0.99$
% of distance in the NW quadrant	38.55 ± 2.345 vs. 36.08 ± 2.827	$t_{61} = 0.4449$	$P > 0.99$
**Recall – D15**			
Escape latency	11.60 ± 1.769 vs. 24.40 ± 2.428	$r = -75.16$	*****$P < 0.001$**
Distance	2.036 ± 0.335 vs. 4.336 ± 0.5421	$r = -60.85$	****$P = 0.003$**
Mean speed	0.171 ± 0.007 vs. 0.1628 ± 0.008	$r = 11.49$	$P > 0.99$
**Flexibility – D16**			
Escape latency	19.18 ± 2.341 vs. 31.62 ± 3.078	$t_{61} = 4.620$	*****$P < 0.001$**
Distance	3.344 ± 0.4634 vs. 5.815 ± 0.7063	$t_{61} = 4.300$	*****$P < 0.001$**
Mean speed	0.1676 ± 0.0084 vs. 0.1656 ± 0.0077	$t_{61} = 0.1861$	$P > 0.99$
**Latency (Learning)**			
Day 17	10.06 ± 1.679 vs. 21.70 ± 2.528	$t_{61} = 4.323$	*****$P < 0.001$**
Day 18	5.734 ± 0.4429 vs. 13.74 ± 1.638	$t_{61} = 2.975$	***$P = 0.04$**
Day 19	6.089 ± 0.4432 vs. 9.021 ± 0.7117	$t_{61} = 1.090$	$P > 0.99$
**Distance (Learning)**			
Day 17	1.904 ± 0.3585 vs. 3.458 ± 0.4989	$t_{61} = 2.706$	$P = 0.10$
Day 18	1.030 ± 0.0992 vs. 2.144 ± 0.2601	$t_{61} = 1.939$	$P = 0.79$
Day 19	1.012 ± 0.1030 vs. 1.227 ± 0.1298	$t_{61} = 0.3741$	$P > 0.99$
**Speed (Learning)**			
Day 17	0.1756 ± 0.0063 vs. 0.1529 ± 0.0078	$t_{61} = 2.146$	$P = 0.48$
Day 18	0.1768 ± 0.0059 vs.	$t_{61} = 2.221$	$P = 0.40$

		0.1534 ± 0.0062		
	Day 19	0.1570 ± 0.0085 vs. 0.1363 ± 0.0076	t_{61} = 1.964	P = 0.75
NaCl/vitamin C vs. 5-FU/vitamin C	**Initiation – D8**			
	Escape latency	44.16 ± 2.823 vs. 49.66 ± 2.773	r = -22.05	P > 0.99
	Distance	6.847 ± 0.5830 vs. 7.223 ± 0.5311	r = -6.955	P > 0.99
	Mean speed	0.1579 ± 0.0078 vs. 0.1437 ± 0.0064	r = 28.55	P > 0.99
	**Latency (Learning)**			
	Day 9	43.58 ± 2.836 vs. 43.31 ± 2.798	t_{61} = 0.0680	P > 0.99
	Day 10	29.43 ± 2.854 vs. 42.36 ± 2.980	t_{61} = 3.332	***P = 0.02**
	Day 11	21.94 ± 2.658 vs. 30.12 ± 2.930	t_{61} = 2.058	P = 0.60
	Day 12	15.15 ± 2.555 vs. 22.45 ± 2.425	t_{61} = 1.836	P > 0.99
	**Distance (Learning)**			
	Day 9	7.803 ± 0.5633 vs. 7.224 ± 0.5731	t_{61} = 0.7870	P > 0.99
	Day 10	5.105 ± 0.5714 vs. 6.816 ± 0.6045	t_{61} = 2.327	P = 0.30
	Day 11	4.043 ± 0.5981 vs. 5.469 ± 0.6622	t_{61} = 1.939	P = 0.79
	Day 12	2.935 ± 0.5841 vs. 3.931 ± 0.5236	t_{61} = 1.354	P > 0.99
	**Speed (Learning)**			
	Day 9	0.1744 ± 0.0057 vs. 0.1581 ± 0.0063	t_{61} = 2.635	P > 0.99
	Day 10	0.1665 ± 0.0061 vs. 0.1517 ± 0.0063	t_{61} = 1.410	P > 0.99
	Day 11	0.1623 ± 0.0070 vs. 0.1596 ± 0.0088	t_{61} = 0.2641	P > 0.99
	Day 12	0.1612 ± 0.0076 vs. 0.1552 ± 0.0087	t_{61} = 0.5738	P > 0.99
	**Probe test – D12**			
	% of time spent in NW quadrant	32.00 ± 4.936 vs. 24.91 ± 2.937	t_{61} = 1.171	P > 0.99

	% of distance in the NW quadrant	30.55 ± 4.559 vs. 25.18 ± 2.600	$t_{61} = 0.9489$	*P* > 0.99
	**Recall – D15**			
	Escape latency	12.25 ± 1.692 vs. 25.64 ± 2.577	*r* = -70.26	********P* < 0.001**
	Distance	2.391 ± 0.397 vs. 4.929 ± 0.6204	*r* = -61.23	*******P* = 0.003**
	Mean speed	0.171 ± 0.007 vs. 0.1763 ± 0.0072	*r* = -4.943	*P* > 0.99
	**Flexibility – D16**			
	Escape latency	17.43 ± 1.886 vs. 28.06 ± 2.754	$t_{61} = 3.867$	*******P* = 0.002**
	Distance	3.140 ± 0.4274 vs. 5.308 ± 0.7630	$t_{61} = 3.696$	*******P* = 0.003**
	Mean speed	0.1631 ± 0.0082 vs. 0.1592 ± 0.01031	$t_{61} = 0.3625$	*P* > 0.99
	**Latency (Learning)**			
	Day 17	9.614 ± 0.9765 vs. 21.78 ± 2.851	$t_{61} = 4.425$	********P* < 0.001**
	Day 18	7.057 ± 0.6315 vs. 13.94 ± 2.166	$t_{61} = 2.505$	*P* = 0.19
	Day 19	5.948 ± 0.4221 vs. 9.373 ± 0.9790	$t_{61} = 1.246$	*P* > 0.99
	**Distance (Learning)**			
	Day 17	1.782 ± 0.2158 vs. 3.406 ± 0.5729	$t_{61} = 2.767$	*P* = 0.09
	Day 18	1.190 ± 0.1304 vs. 2.167 ± 0.3700	$t_{61} = 1.664$	*P* > 0.99
	Day 19	0.9513 ± 0.1006 vs. 1.215 ± 0.1290	$t_{61} = 0.4488$	*P* > 0.99
	**Speed (Learning)**			
	Day 17	0.1720 ± 0.0059 vs. 0.1502 ± 0.0090	$t_{61} = 2.026$	*P* = 0.65
	Day 18	0.1589 ± 0.0071 vs. 0.1503 ± 0.0060	$t_{61} = 0.7904$	*P* > 0.99
	Day 19	0.1465 ± 0.0070 vs. 0.1307 ± 0.0051	$t_{61} = 1.467$	*P* > 0.99
NaCl/Qiseng® vs.	**Initiation – D8**			
	Escape latency	39.85 ± 3.696 vs. 40.61 ± 3.548	*r* = -8.685	*P* > 0.99

5-FU/Qiseng®	Distance	5.996 ± 0.6525 vs. 3.118 ± 0.6588	r = -4.721	P > 0.99
	Mean speed	0.1313 ± 0.0089 vs. 0.1330 ± 0.0088	r = -8.296	P > 0.99
	**Latency (Learning)**			
	Day 9	33.65 ± 3.395 vs. 44.87 ± 2.897	t_{61} = 2.808	P = 0.08
	Day 10	23.57 ± 2.861 vs. 42.20 ± 2.945	t_{61} = 4.715	********P* < 0.001**
	Day 11	18.54 ± 2.408 vs. 29.53 ± 3.318	t_{61} = 2.753	P = 0.09
	Day 12	11.36 ± 1.770 vs. 21.39 ± 2.876	t_{61} = 2.512	P = 0.18
	**Distance (Learning)**			
	Day 9	4.575 ± 0.5722 vs. 6.755 ± 0.5277	t_{61} = 2.953	******P* = 0.05**
	Day 10	3.307 ± 0.4339 vs. 6.219 ± 0.4770	t_{61} = 3.944	*******P* = 0.001**
	Day 11	2.612 ± 0.404 vs. 4.710 ± 0.6184	t_{61} = 2.842	P = 0.07
	Day 12	1.903 ± 0.3973 vs. 2.919 ± 0.3955	t_{61} = 1.375	P > 0.99
	**Speed (Learning)**			
	Day 9	0.1360 ± 0.0079 vs. 0.1473 ± 0.0079	t_{61} = 1.076	P > 0.99
	Day 10	0.1388 ± 0.0062 vs. 0.1469 ± 0.0064	t_{61} = 0.7748	P > 0.99
	Day 11	0.1350 ± 0.0081 vs. 0.1513 ± 0.0081	t_{61} = 1.548	P > 0.99
	Day 12	0.1466 ± 0.0080 vs. 0.1424 ± 0.0083	t_{61} = 0.4032	P > 0.99
	**Probe test – D12**			
	% of time spent in NW quadrant	42.70 ± 4.856 vs. 30.67 ± 6.039	t_{61} = 1.978	P = 0.79
	% of distance in the NW quadrant	41.90 ± 4.743 vs. 30.58 ± 5.508	t_{61} = 0.1994	P = 0.76
	**Recall – D15**			
	Escape latency	6.488 ± 0.7373 vs. 21.07 ± 2.550	r = -87.18	********P* < 0.001**

Distance	1.045 ± 0.15 vs. 2.913 ± 0.333	$r = -73.16$	***$P < 0.001$
Mean speed	0.159 ± 0.012 vs. 0.1537 ± 0.0089	$r = -1.069$	$P > 0.99$
**Flexibility – D16**			
Escape latency	20.18 ± 2.937 vs. 31.30 ± 3.095	$t_{61} = 4.027$	***$P < 0.001$
Distance	3.704 ± 0.6476 vs. 5.279 ± 0.6550	$t_{61} = 2.673$	$P = 0.11$
Mean speed	0.1604 ± 0.0086 vs. 0.1624 ± 0.0085	$t_{61} = 0.1847$	$P > 0.99$
**Latency (Learning)**			
Day 17	6.580 ± 0.7246 vs. 22.21 ± 2.941	$t_{61} = 5.661$	***$P < 0.001$
Day 18	5.455 ± 0.4858 vs. 12.60 ± 1.870	$t_{61} = 2.588$	$P = 0.15$
Day 19	4.685 ± 0.3472 vs. 7.710 ± 0.6386	$t_{61} = 1.096$	$P > 0.99$
**Distance (Learning)**			
Day 17	1.129 ± 0.1614 vs. 3.743 ± 0.5717	$t_{61} = 4.435$	***$P < 0.001$
Day 18	0.8950 ± 0.0948 vs. 2.014 ± 0.4313	$t_{61} = 1.899$	$P = 0.87$
Day 19	0.7647 ± 0.0722 vs. 1.113 ± 0.1204	$t_{61} = 0.5916$	$P > 0.99$
**Speed (Learning)**			
Day 17	0.1626 ± 0.0065 vs. 0.1664 ± 0.0074	$t_{61} = 0.3444$	$P > 0.99$
Day 18	0.1597 ± 0.0075 vs. 0.1460 ± 0.0083	$t_{61} = 1.265$	$P > 0.99$
Day 19	0.1578 ± 0.0077 vs. 0.1381 ± 0.0073	$t_{61} = 1.819$	$P > 0.99$

Note. Different items were analyzed in the open field test, the actimetry test, the light/dark box test, the elevated plus maze test, the anhedonia test, the tail suspension test, the forced swimming test and the Morris water maze test in controls and 5-FU groups treated with placebo, vitamin C or Qiseng®. Behavioral data are expressed as mean ± SEM ($n = 10 - 12$). When the data followed a normal distribution, one-way or two-way (in case of repeated measures) ANOVA was performed, with a Bonferroni test used for post-hoc analyses (indicated as t followed by P value). In the absence of normal data distribution, a non parametric Kruskal-Wallis test was performed, with a Dunn's test for post-hoc analyses (indicated as r followed by P value). *5-FU: 5-fluorouracil.*

Table S3. Impact of chemotherapy injection on plasma pro-inflammatory, pluripotent, chemotactic and leukocyte growth cytokines.

		placebo<br>NaCl vs. 5-FU	vitamin C<br>NaCl vs. 5-FU	Qiseng®<br>NaCl vs. 5-FU
**Proinflammatory cytokines**				
IL-1α	Mean ± SEM	40.8 ± 10.96 vs. 25.83 ± 10.5	40.93 ± 5.59 vs. 18.16 ± 3.78	28.34 ± 6.62 vs. 30.28 ± 6.32
	P. value	ns. (P = 0.07)	****P = 0.001**	ns.
IL-1β	Mean ± SEM	3.84 ± 0.85 vs. 4.66 ± 0.51	2.52 ± 0.15 vs. 3.13 ± 0.36	3.38 ± 0.66 vs. 3.5 ± 0.54
	P. value	ns. (P = 0.06)	ns. (P = 0.05)	ns.
IL-12p70	Mean ± SEM	8.21 ± 3.19 vs. 15.29 ± 2.77	4.54 ± 1.44 vs. 3.39 ± 0.90	8.18 ± 1.99 vs. 8.71 ± 2.1
	P. value	***P = 0.03**	ns.	ns.
TNFα	Mean ± SEM	1.53 ± 0.80 vs. 4.37 ± 1.33	0.52 ± 0.25 vs. 0.85 ± 0.39	1.39 ± 0.42 vs. 1.54 ± 0.45
	P. value	****P = 0.009**	ns.	ns.
**Pluripotent cytokines**				
IL-2	Mean ± SEM	7.19 ± 1.39 vs. 22.82 ± 7.5	6.3 ± 0.6 vs. 5.71 ± 0.16	7.0 ± 0.77 vs. 6.8 ± 0.45
	P. value	***P = 0.04**	ns.	ns.
IL-4	Mean ± SEM	5.21 ± 1.17 vs. 8.26 ± 1.14	4.08 ± 0.37 vs. 7.71 ± 3.28	5.29 ± 0.68 vs. 5.32 ± 0.61
	P. value	****P = 0.001**	ns. (P = 0.07)	ns.
IL-6	Mean ± SEM	9.91 ± 1.71 vs. 36.31 ± 7.42	9.78 ± 1.22 vs. 30.0 ± 16.48	12.62 ± 2.78 vs. 12.57 ± 1.68
	P. value	*****P < 0.001**	***P = 0.02**	ns.
IL-17	Mean ± SEM	8.13 ± 1.57 vs. 27.95 ± 7.16	6.13 ± 0.38 vs. 14.93 ± 8.02	7.83 ± 0.79 vs. 8.82 ± 0.88
	P. value	*****P < 0.001**	ns. (P = 0.06)	ns.
**Chemotactic cytokines**				
MCP-1	Mean ± SEM	37.16 ± 3.61 vs. 235 ± 65.54	38.14 ± 2.06 vs. 142.6 ± 70.7	42.42 ± 5.3 vs. 61.92 ± 15.83
	P. value	*****P < 0.001**	*****P < 0.001**	ns. (P = 0.08)
RANTES	Mean ± SEM	18.29 ± 2.91 vs. 11.52 ± 2.78	19.52 ± 2.7 vs. 10.08 ± 1.13	15.64 ± 2.01 vs. 12.54 ± 1.52
	P. value	***P = 0.05**	***P = 0.02**	ns.
**Leukocyte growth cytokines**				
IL-3	Mean ± SEM	4.45 ± 0.83 vs. 5.17 ± 0.62	3.4 ± 0.26 vs. 3.28 ± 0.18	4.09 ± 0.42 vs. 3.8 ± 0.36
	P. value	ns. (P = 0.07)	ns.	ns.
GMCSF	Mean ± SEM	12.07 ± 1.77 vs. 18.13 ± 2.52	10.52 ± 2.22 vs. 15.54 ± 4.05	11.03 ± 1.95 vs. 12.58 ± 3.11
	P. value	ns. (P = 0.06)	ns.	ns.

Note. Quantification of plasmatic cytokines from controls and 5-FU groups, as determined by ELISA. All data are expressed in pg/ml. Data are expressed as mean ± SEM (n = 6 - 10, Mann-Whitney). *5-FU: 5-fluorouracil, GMCSF: Granulocyte Macrophage Colony-Stimulating Factor, IL: Interleukine, MCP-1: Monocyte Chemotactic Protein-1, TNF-α: Tumor Necrosis Factor-α, RANTES: Regulated on Activation Normal T cell Expressed and Secreted.*

References

1. Klindworth, A.; Pruesse, E.; Schweer, T.; Peplies, J.; Quast, C.; Horn, M.; Glöckner, F.O. Evaluation of general 16S ribosomal RNA gene PCR primers for classical and next-generation sequencing-based diversity studies. *Nucleic acids research* **2013**, *41*, e1, doi:10.1093/nar/gks808.
2. Fierer, N.; Jackson, J.A.; Vilgalys, R.; Jackson, R.B. Assessment of soil microbial community structure by use of taxon-specific quantitative PCR assays. *Applied and environmental microbiology* **2005**, *71*, 4117-4120, doi:10.1128/aem.71.7.4117-4120.2005.
3. Queipo-Ortuño, M.I.; Seoane, L.M.; Murri, M.; Pardo, M.; Gomez-Zumaquero, J.M.; Cardona, F.; Casanueva, F.; Tinahones, F.J. Gut microbiota composition in male rat models under different nutritional status and physical activity and its association with serum leptin and ghrelin levels. *PLoS One* **2013**, *8*, e65465, doi:10.1371/journal.pone.0065465.
4. Mühling, M.; Woolven-Allen, J.; Murrell, J.C.; Joint, I. Improved group-specific PCR primers for denaturing gradient gel electrophoresis analysis of the genetic diversity of complex microbial communities. *The ISME journal* **2008**, *2*, 379-392, doi:10.1038/ismej.2007.97.
5. Hermann-Bank, M.L.; Skovgaard, K.; Stockmarr, A.; Larsen, N.; Mølbak, L. The Gut Microbiotassay: a high-throughput qPCR approach combinable with next generation sequencing to study gut microbial diversity. *BMC genomics* **2013**, *14*, 788, doi:10.1186/1471-2164-14-788.
6. Bindels, L.B.; Beck, R.; Schakman, O.; Martin, J.C.; De Backer, F.; Sohet, F.M.; Dewulf, E.M.; Pachikian, B.D.; Neyrinck, A.M.; Thissen, J.P.; et al. Restoring specific lactobacilli levels decreases inflammation and muscle atrophy markers in an acute leukemia mouse model. *PLoS One* **2012**, *7*, e37971, doi:10.1371/journal.pone.0037971.
